# *Gyrodactylus salinae *n. sp. (Platyhelminthes: Monogenea) infecting the south European toothcarp *Aphanius fasciatus *(Valenciennes) (Teleostei, Cyprinodontidae) from a hypersaline environment in Italy

**DOI:** 10.1186/1756-3305-4-100

**Published:** 2011-06-09

**Authors:** Giuseppe Paladini, Tine Huyse, Andrew P Shinn

**Affiliations:** 1 Institute of Aquaculture, University of Stirling, Stirling, FK9 4LA, Scotland, UK; 2 Katholieke Universiteit Leuven, Laboratory of Animal Diversity and Systematics, Ch. Deberiotstraat 32, B-3000 Leuven, Belgium

## Abstract

**Background:**

Historically, non-native species of *Gambusia *(Poeciliidae) have been used to control larval stages of the Asian tiger mosquito, *Stegomyia albopicta *Reinert, Harbach *et *Kitching, 2004 throughout Italy. The potential utility of indigenous populations of *Aphanius fasciatus *(Valenciennes) (Teleostei: Cyprinodontidae) as an appropriate alternative biological control is currently being explored. A sub-sample of ten fish collected from Cervia Saline, Italy (salinity 65 ppt; 30°C) to assess their reproductive capability in captivity, harboured a moderate infection of *Gyrodactylus *von Nordmann, 1832 (Platyhelminthes, Monogenea). A subsequent morphological and molecular study identified this as being a new species.

**Results:**

*Gyrodactylus salinae *n. sp. is described from the skin, fins and gills of *A. fasciatus*. Light and scanning electron microscopical (SEM) examination of the opisthaptoral armature and their comparison with all other recorded species suggested morphological similarities to *Gyrodactylus rugiensoides *Huyse *et *Volckaert, 2002 from *Pomatoschistus minutus *(Pallas). Features of the ventral bar, however, permit its discrimination from *G. rugiensoides*. Sequencing of the nuclear ribosomal DNA internal transcribed spacers 1 and 2 and the 5.8S rRNA gene and a comparison with all species listed in GenBank confirmed they are unique and represent a new species (most similar to *Gyrodactylus anguillae *Ergens, 1960, 8.3% pair-wise distance based on 5.8S+ITS2). This represents the first species of *Gyrodactylus *to be described from *Aphanius *and, to date, has the longest ITS1 (774 bp) sequenced from any *Gyrodactylus*. Additional sampling of Cervia Saline throughout the year, found *G. salinae *n. sp. to persist in conditions ranging from 35 ppt and 5°C in December to 65 ppt and 30°C in July, while in captivity a low level of infection was present, even in freshwater conditions (0 ppt).

**Conclusions:**

The ability of *G. salinae *n. sp. to tolerate a wide range of salinities and temperatures shows its potential to readily adapt to several environmental conditions. These findings, together with the fact that *A. fasciatus *is a protected species and is considered as a biological control organism, necessitate further studies on the ecology and virulence of *G. salinae *n. sp.

## Background

Toothcarp is a colloquial term used to describe members of the order Cyprinodontiformes, which comprises ten fish families, namely Anablepidae, Aplocheilidae, Cyprinodontidae, Fundulidae, Goodeidae, Nothobranchiidae, Poeciliidae, Profundulidae, Rivulidae and Valenciidae [1]. Within the family Cyprinodontidae, the south European toothcarp *Aphanius fasciatus *(Valenciennes) is one of the more commonly occurring neritic species within the Mediterranean and is characteristically known to be an eurythermic and euryhaline fish species, for its ability to tolerate a wide range of temperatures (5-39°C) and salinities (0-180 ppt), respectively [2]. *Aphanius fasciatus *has a widespread distribution along the Italian coastline, principally in brackish waters [3], although increasing anthropogenic activity has caused a general decline in numbers. For this reason, *A. fasciatus *is considered to be a species that is "dying out" and as such, is listed under Appendix III "Protected Fauna Species" after the Bern Convention on the conservation of European wildlife and natural habitats, and under Annex II of the Council Directive 92/43/EEC on the conservation of natural habitats and of wild fauna and flora in the European Community [4]. It has recently been used as a biological control against the larvae of the Asian tiger mosquito *Stegomyia albopicta *(= *Aedes albopictus*) Reinert, Harbach *et *Kitching, 2004 which are vectors for a range of human infectious diseases, including Chikungunya fever, dengue fever, West Nile fever and yellow fever [5-7].

During a recent research study investigating the artificial reproduction of *A. fasciatus *in captivity as part of a large scale restocking and mosquito control initiative in Italy [8,9], several specimens collected, under licence, on a number of occasions were found to harbour an infection of *Gyrodactylus *von Nordmann, 1832, principally on the skin and fins and, to a lesser degree, on the gills. Infected tissues were removed from the moribund fish and subsequently sent to the Institute of Aquaculture, University of Stirling (Scotland, UK) for identification. Given the importance of *A. fasciatus *as a protected species and its utility as an alternative indigeneous biological control agent to the introduced *Gambusia *spp. (Poeciliidae), more information on its *Gyrodactylus *fauna was needed.

## Methods

### Specimens collection and preparation

A total of ten adult *A. fasciatus *(total length 4-7 cm; weight ~3 g) was collected during July 2008 from isolated pools in Cervia Saline, located in the Emilia Romagna region in northern Italy, and fixed in 70% ethanol. All ten specimens collected from the drying, landlocked pools were moribund individuals, a consequence of reduced water availability and increased algal growth. The skin, fins and gills of each fish were subsequently screened for metazoan parasites using an Olympus SZ40 stereomicroscope at ×4 magnification. Specimens of *Gyrodactylus *were removed using mounted triangular surgical needles. All ten fish were infected but given the condition of the fish on collection, the intensity of infection can only be estimated at between 10-30 parasites per fish.

The alcohol-fixed parasites were subsequently rinsed in distilled water and representatives prepared as whole mounts using ammonium picrate glycerine following the procedure detailed by Malmberg [10]. Additional specimens had their opisthaptors removed using a scalpel, which were then individually subjected to proteolytic digestion on glass slides, as described in Paladini *et al*. [11]. The largely tissue-free opisthaptoral hook preparations were then mounted in ammonium picrate glycerine using an 18 × 18 mm coverslip, the edges of which were sealed with a commercial brand of nail varnish. The corresponding body of each specimen of *Gyrodactylus *was fixed in 90% ethanol for subsequent molecular characterisation.

For scanning electron microscopy (SEM), single specimens of *Gyrodactylus *were subjected to full proteolytic digestion on 11 mm round glass coverslips to obtain tissue-free attachment hooks. Each digestion took approximately 60 min, with 3 μl of digestion solution being added every 10 min, punctuated by the addition of 5 μl distilled water for five times to remove digested tissue residues and debris. For each step, the digested hook preparations were placed in a Petri dish to protect them from extraneous dust and placed in an incubator at 55°C to help digestion, followed by a final incubation at 40°C overnight to dry. The position of hooks on each coverslip was subsequently marked using tiny, adhesive, triangular labels positioned using forceps under an Olympus BX51 compound microscope at ×10 magnification. The coverslips were then attached onto aluminium stubs with bi-adhesive round labels, sputter-coated with gold using an Edwards S150B sputter coater and then examined using a JEOL JSM5200 SEM operating at an accelerating voltage of 10 kv.

### Morphological analysis

For the morphological study, images of the opisthaptoral hard parts and the male copulatory organ (MCO) were captured at magnifications of ×40 and ×100 oil immersion using MRGrab 1.0.0.4 (Carl Zeiss Vision GmbH, 2001) software and a Zeiss AxioCam MRc digital camera mounted on an Olympus BX51 compound microscope, using a ×0.75 interfacing lens. Drawings of the taxonomic features were made from the captured images. Each *Gyrodactylus *specimen was subjected to morphometric analysis taking a total of 27 point-to-point measurements on the opisthaptoral hooks using a JVC KY-F30B 3CCD video camera mounted on an Olympus BH2 microscope using a ×2.5 interfacing lens at ×100 oil immersion and the KS300 (ver.3.0) (Carl Zeiss Vision GmbH, 1997) image analysis software, combined with the specific macro for gyrodactylids, Point-R (Bron & Shinn, University of Stirling). The point-to-point hook measurements for the specimens are given in micrometres as the mean ± 1 standard deviation followed by the range in parentheses, and follow those described in Shinn *et al*. [12], plus three additional measurements of the dorsal bar (total length, width and attachment point length).

The gyrodactylid material prepared from *A. fasciatus *was compared to type material of *Gyrodactylus rugiensoides *Huyse *et *Volckaert, 2002 (paratypes acc. nos. BMNH 2002.2.14.2-3), a species with morphologically similar marginal hooks, held in the Parasitic Worms collection at The Natural History Museum, London, UK. In addition, type material of the six *Gyrodactylus *species known to parasitise cyprinodontid hosts, held in the U.S. National Parasite Collection, Beltsville, Maryland, USA, was examined, the marginal hooks re-drawn and compared to the new specimens collected from *A. fasciatus*. These are *Gyrodactylus cyprinodontis *Mizelle *et *Kritsky, 1967 (holotype and paratype acc. no. USNPC 62951), *Gyrodactylus hargisi *Williams *et *Rogers, 1971 (paratypes acc. no. USNPC 71760), *Gyrodactylus mobilensis *Williams *et *Rogers, 1971 (paratypes acc. no. USNPC 71762), *Gyrodactylus nevadensis *Mizelle *et *Kritsky, 1967 (holotype and paratype acc. no. USNPC 62954), *Gyrodactylus saratogensis *Mizelle *et *Kritsky, 1967 (paratype acc. no. USNPC 62956) and *Gyrodactylus tularosae *Kritsky *et *Stockwell, 2005 (paratype acc. no. USNPC 94780).

### Molecular analysis

The bodies of 2 specimens were individually transferred to a 0.2 ml microcentrifuge tube containing 5 μl of milli-Q water and digested by the addition of 5 μl of lysis solution consisting of 1×PCR buffer (Eurogentec, Seraing, Belgium), 0.45% (v/v) Tween 20, 0.45% (v/v) NP 40 and 60 μg/ml of proteinase K (Sigma, Poole, UK). The samples were incubated at 65°C for 25 min, followed by 10 min at 95°C to inactivate the proteinase. The primer pairs ITS1A (5'-GTAACAAGGTTT CCGTAGGTG-3') and ITS2 (5'-TCCTCCGCTTAGTGATA- 3') [13] were used to amplify a fragment spanning the 3' end of the 18S rRNA gene, the internal transcribed spacer 1 (ITS1), the 5.8S rRNA gene, ITS2, and the 5' end of the 28S rRNA gene. The amplification reactions (20 μl) consisted of 1×PCR buffer, 1.5 mM MgCl_2 _(Eurogentec, Seraing, Belgium), 200 μM of each dNTP (Amersham Pharmacia Biotech, Uppsala, Sweden), 1 μM of each primer (Eurogentec, Seraing, Belgium), 2 μl lysate, 1 unit *Taq *polymerase (Eurogentec, Seraing, Belgium) and milli-Q water. The mixtures were heated for 4 min at 96°C and subjected to 35 cycles of 1 min at 95°C, 1 min at 50°C and 2 min at 72°C, followed by a final extension at 72°C for 7 min. The PCR products were visualised using ethidium bromide on a 1.2% agarose gel. The products were then purified by means of GFX columns according to the manufacturer's instructions (Amersham Pharmacia Biotech, Uppsala, Sweden). Both DNA strands were sequenced using a Big Dye Chemistry Cycle Sequencing Kit (version 1.1) in a 3130 DNA Analyzer (Applied Biosystems, Belgium). The PCR primers and 2 internal primers, ITS1R (5'-ATTT GCGTTCGAGAGACCG-3') and ITS2F (5'-TGGTGGATCA CTCGGCTCA-3') [14], were used for sequencing.

The obtained sequences were subjected to a BLAST search (available at http://www.ncbi.nih.gov/BLAST/) to identify similar sequences among other species of *Gyrodactylus *in GenBank [15]. The sequences with the highest similarity to our sequences were downloaded and the 5.8S and ITS2 fragments were aligned in Clustal W implemented in MEGA 4 [16]. Pair-wise genetic distances were computed in MEGA 4 according to the evolutionary model that was selected by jModelTest 0.1.1 [17]. The sequences were also scanned for repeat elements using the program Tandem Repeats Finder [18].

## Results

### *Gyrodactylus salinae *n. sp

#### Type host

*Aphanius fasciatus *(Valenciennes), Cyprinodontidae ("South European toothcarp", "nono").

#### Site of infection

Skin, fins and occasionally gills.

#### Type locality

Cervia Saline, Emilia Romagna region, Italy (44°14'N, 12°20'E).

#### Environmental conditions under which specimens were collected

Salinity and temperature in July 2008, 65 ppt and 30°C, respectively.

#### Type material

Fifteen specimens were studied for light microscopy. Holotype (acc. no. BMNH 2011.5.19.1) and four paratypes (acc. nos. BMNH 2011.5.19.2-5) are deposited in the parasitic worm collection at The Natural History Museum, London. Additionally, three paratypes (acc. no. M-521) are deposited in the gyrodactylid collection held at the Institute of Parasitology, Academy of Sciences of the Czech Republic, České Budĕjovice; four paratypes (acc. nos. AHC 35118-35121) are deposited in the Australian Helminthological Collection (AHC) of The South Australian Museum (SAMA), North Terrace, Adelaide; and three paratypes (acc. nos. USNPC 104748-104750) are deposited in the United States National Parasite Collection, Beltsville, Maryland, USA.

#### Molecular sequence data

The sequence fragment of approximately 1379 bp encoding partial 18S (32 bp), ITS1 (774 bp), 5.8S (158 bp), ITS2 (402 bp) and partial 28S (13 bp) is deposited in GenBank under accession no. JF950559.

#### General

A species profile including host and taxonomic details is provided on the on-line databases http://www.gyrodb.net[19] and http://www.monodb.org[20].

#### Etymology

Named after the Italian generic name for a hypersaline water body *i.e*. "salina" (= saline in English) and the broad salinity tolerance exhibited by this species of *Gyrodactylus*.

### Morphological description (Figure 1, 2, 3; Table 1)

**Figure 1:**
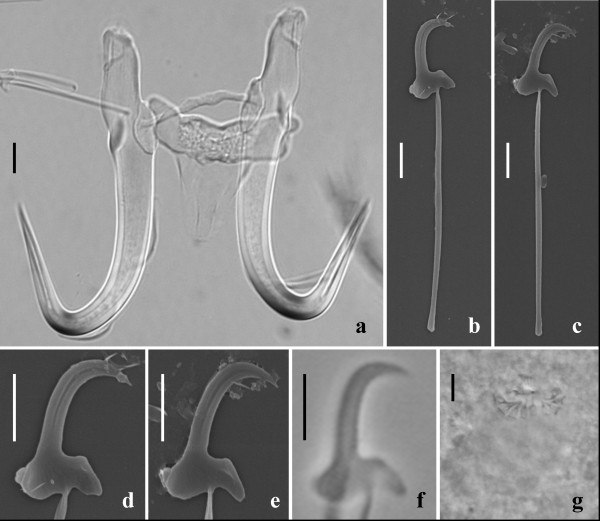
***Gyrodactylus salinae *n. sp. from the south European toothcarp *Aphanius fasciatus *(Valenciennes) from Cervia Saline, Italy**. **a **- light micrograph of the opisthaptoral central hook complex showing the hamuli, the dorsal bar and the ventral bar (ventral view); **b**, **c **- scanning electron micrographs (SEM) of the marginal hooks; **d**, **e **- SEM of the marginal hook sickles; **f - **light micrograph of a marginal hook sickle; **g - **light micrograph of the male copulatory organ (MCO) bearing one principal spine and nine small spines in a single row. Scale bars: **a **= 5 μm; **b**-**g **= 3 μm.

**Figure 2:**
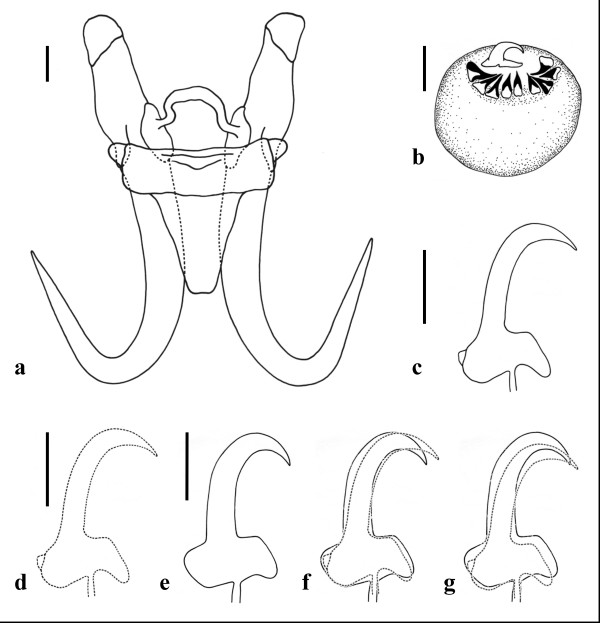
**Drawings of the opisthaptoral hard parts and male copulatory organ (MCO) of *Gyrodactylus salinae *n. sp. from the south European toothcarp *Aphanius fasciatus *(Valenciennes) from Cervia Saline, Italy**. **a **- opisthaptoral central hook complex; **b **- MCO; **c**, **d **- marginal hook sickles; **e **- marginal hook sickle of *Gyrodactylus rugiensoides *Huyse *et *Volckaert, 2002 from *Pomatoschistus minutus *(Pallas) collected from Texel, The Netherlands (re-drawn from the paratype 2002.2.14.2); **f **- a size invariant overlay of the marginal hook sickles of *G. salinae *n. sp. (broken line) with *G. rugiensoides *(solid line); **g **- a size variant overlay of the marginal hook sickles of *G. salinae *n. sp. (broken line) with *G. rugiensoides *(solid line). Scale bars: **a**, **b **= 5 μm; **c**-**e **= 3 μm.

**Figure 3:**
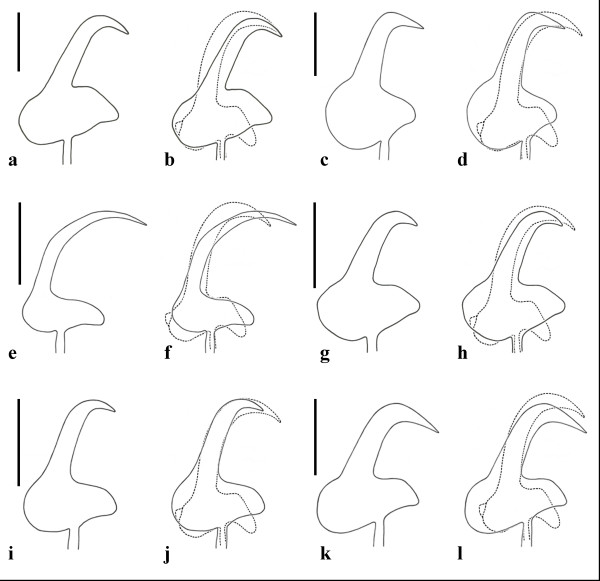
**A size invariant comparison of the marginal hook sickle of *Gyrodactylus salinae *n. sp. (broken line) from the south European toothcarp *Aphanius fasciatus *(Valenciennes) from Cervia Saline (Italy), with the other six *Gyrodactylus *species previously recorded infecting cyprinodontid hosts**. **a **- *Gyrodactylus cyprinodontis *Mizelle *et *Kritsky, 1967; **b **- overlay of *G. salinae *n. sp. with *G. cyprinodontis *(solid line); **c **- *Gyrodactylus hargisi *Williams *et *Rogers, 1971; **d **- overlay of *G. salinae *n. sp. with *G. hargisi *(solid line); **e **- *Gyrodactylus mobilensis *Williams *et *Rogers, 1971; **f **- overlay of *G. salinae *n. sp. with *G. mobilensis *(solid line); **g **- *Gyrodactylus nevadensis *Mizelle *et *Kritsky, 1967; **h **- overlay of *G. salinae *n. sp. with *G. nevadensis *(solid line); **i **- *Gyrodactylus saratogensis *Mizelle *et *Kritsky, 1967; **j **- overlay of *G. salinae *n. sp. with *G. saratogensis *(solid line); **k **- *Gyrodactylus tularosae *Kritsky *et *Stockwell, 2005; **l **- overlay of *G. salinae *n. sp. with *G. tularosae *(solid line). Scale bars = 3 μm.

**Table 1:** Morphological measurements (mean ± 1 standard deviation followed by the range in parentheses) of *Gyrodactylus salinae *n. sp. from *Aphanius fasciatus *(Valenciennes) collected from Cervia Saline, Italy, compared with those of *Gyrodactylus rugiensoides *Huyse *et *Volckaert, 2002 from *Pomatoschistus minutus *(Pallas) collected from Texel, The Netherlands (paratypes acc. nos. BMNH 2002.2.14.2-3)

Variable	***Gyrodactylus salinae *n. sp**. (n = 15)	*Gyrodactylus rugiensoides *Huyse *et *Volckaert, 2002 (n = 2)
Total body length	447 ± 63.3 (375-575)	787.5 (700-875)
Total body width	116 ± 26.7 (88-163)	159 (155-162.5)
Opisthaptor length × width	75 ± 8.9 (60-88) × 80 ± 5.7 (70-88)	112.5 (110-115) × 147.5 (145-150)
Anterior pharynx bulb length × width	18.8 ± 3.1 (15.0-24.5) × 22.1 ± 2.4 (19.5-27.2)	32.9 (30.1-35.7) × 48.3 (46.8-49.7)
Posterior pharynx bulb length × width	8.1 ± 0.7 (6.6-8.8) × 24.6 ± 2.4 (21.8-29.0)	34.1 (27.7-40.5) × 78.4 (76.5-80.4)
MCO length × width	14.4 ± 2.1 (11.9-18.5) × 12.7 ± 2.7 (10.1-17.6)^1^	14.8 (13.6-15.9) × 15.1 (14.0-16.1)
**Hamulus (H)**		
H aperture	17.9 ± 0.8 (17.0-19.6)	18.0 (17.5-18.5)
H proximal shaft width	7.0 ± 0.6 (6.3-8.1)	8.1 (7.6-8.6)
H point length	24.8 ± 0.5 (23.9-25.9)	29.9 (29.3-30.5)
H distal shaft width	4.1 ± 0.4 (3.2-4.6)	5.4 (5.3-5.4)
H shaft length	31.9 ± 2.9 (28.2-37.3)	33.8 (33.5-34.2)
H inner curve length	3.8 ± 0.6 (2.3-4.6)	3.9 (3.5-4.3)
H aperture angle (°)	36.6 ± 1.2 (33.8-37.9)	32.5 (30.7-34.4)
H point curve angle (°)	12.2 ± 2.3 (7.4-15.4)	10.1 (9.4-10.7)
Inner H aperture angle (°)	41.4 ± 1.3 (39.2-43.5)	38.5 (36.4-40.7)
H root length	16.8 ± 0.9 (14.7-18.3)	19.4 (19.2-19.7)
H total length	51.7 ± 1.6 (48.7-54.6)	58.1 (57.9-58.2)
**Dorsal bar (DB)**		
DB total length	20.9 ± 1.7 (18.1-22.9)	25.6 (23.8-27.4)
DB width	1.9 ± 0.2 (1.6-2.2)	1.8 (1.7-1.9)
DB attachment point length	9.4 ± 0.4 (8.8-9.9)	8.7 (8.6-8.7)
**Ventral bar (VB)**		
VB total width	25.0 ± 1.1 (23.5-26.7)	26.2 (25.3-27.1)
VB total length	22.5 ± 1.6 (19.2-25.5)	20.4 (20.4-20.5)
VB process-to-mid length	3.0 ± 0.5 (2.1-4.0)	2.0 (1.8-2.3)
VB median length	6.2 ± 0.4 (5.6-6.8)	6.2 (5.9-6.6)
VB process length	2.9 ± 0.4 (2.1-3.5)	1.2 (1.1-1.3)
VB membrane length	13.2 ± 1.1 (10.7-15.2)	11.9 (11.8-11.9)
**Marginal hook (MH)**		
MH total length	26.8 ± 0.6 (25.9-27.6)	30.7 (30.7-30.8)
MH shaft length	20.8 ± 0.3 (20.2-21.5)	24.3 (24.1-24.5)
MH sickle length	6.3 ± 0.2 (6.1-6.6)	6.6 (6.3-6.9)
MH sickle proximal width	4.0 ± 0.2 (3.6-4.4)	4.3 (4.2-4.3)
MH toe length	1.8 ± 0.1 (1.6-2.0)	1.7 (1.6-1.7)
MH sickle distal width	3.6 ± 0.2 (3.3-4.0)	3.7 (3.5-3.9)
MH aperture	5.6 ± 0.2 (5.4-6.0)	5.7 (5.6-5.7)
MH instep/arch height	0.6 ± 0.1 (0.5-0.7)	0.5 (0.5-0.6)

Measurements are provided in micrometres and follow those detailed in Shinn *et al*. [12], plus three additional measurements of the dorsal bar. Due to the limited number of *G. rugiensoides *specimens available for study, only the mean and range is presented.

^1^Based on the measurement of 5 specimens.

Based on the measurements of fifteen specimens. Body elongate, 447 (375-575) long, 116 (88-163) wide. Prohaptor with a single pair of cephalic lobes each bearing a gland and a spike sensillum. Pharynx small, anterior pharyngeal bulb 18.8 (15.0-24.5) long, 22.1 (19.5-27.2) wide; posterior pharyngeal bulb 8.1 (6.6-8.8) long, 24.6 (21.8-29.0) wide. Intestinal crura extending beyond the anterior edge of the testes. Presence or absence of excretory bladders not discernible on whole mounts. Opisthaptor ovate, 75 (60-88) long × 80 (70-88) wide. Male copulatory organ (MCO) spherical, 14.4 (11.9-18.5) long × 12.7 (10.1-17.6) wide, armed with one principal spine and 9 small spines in a single row. MCO position variable, usually on the left, posterior to the posterior pharyngeal bulb. Total length of hamuli 51.7 (48.7-54.6); hamulus shaft length 31.9 (28.2-37.3); hamulus point 24.8 (23.9-25.9) long, arising at an angle of 41.4° (39.2-43.5°) (internal measurement) to the shaft of the hamulus; hamulus root 16.8 (14.7-18.3) long. Dorsal bar attachment points on hamuli 9.4 (8.8-9.9) long. Dorsal bar simple, 20.9 (18.1-22.9) long, 1.9 (1.6-2.2) wide irregular and slightly thickened at its mid-point. Ventral bar triangular in approximate dimensions, 22.5 (19.2-25.5) long, 25.0 (23.5-26.7) wide; ventral bar processes prominent, arise tangentially to the extremities of the median portion of the ventral bar proper, 2.9 (2.1-3.5) long; ventral bar membrane long, approximately triangular with a smoothly rounded terminal edge, 13.2 (10.7-15.2) long. Total length of marginal hooks 26.8 (25.9-27.6); marginal hook shaft 20.8 (20.2-21.5) long; marginal hook sickle proper 6.3 (6.1-6.6) long. Sickle shaft approximately perpendicular to the base, very slightly angled forward, proportionately slender, turns at a near right angle into a narrow tip which terminates at a point beyond the perpendicular limit of the toe. Sickle distal width 3.6 (3.3-4.0), proximal width 4.0 (3.6-4.4). Aperture of marginal sickle, open, 5.6 (5.4-6.0) long, inner curve of the sickle approximately rectangular. Sickle base has a flat bridge; triangular toe 1.8 (1.6-2.0) long; prominent rounded heel, tangential to the sickle base, downwardly directed. The sickle heel appears square in dimensions in specimens prepared for light microscopy but SEM images reveal a rounded heel with an additional prominent button for muscle attachment (Figure 1b-e).

### Molecular characterisation

The total fragment (1379 bp) consists of the 3' end of the 18S subunit (32 bp), the ITS1 (774 bp), the 5.8S gene (158 bp), the ITS2 (402 bp) and the 5' end of the 28S subunit (13 bp). Both specimens had identical sequences. *Gyrodactylus salinae *n. sp. appeared most closely related to *Gyrodactylus *species belonging to the *G*. (*Paranephrotus*) and *G*. (*Neonephrotus*) sub-genera (sub-genera according to Malmberg [10]) based on the pair-wise distances (Tamura-Nei gamma corrected distances [21] using the 5.8S-ITS2 fragment, Table 2); it is most closely related to *Gyrodactylus anguillae *Ergens, 1960 (8.3%; acc. no. AB063294) and *Gyrodactylus micropsi *Gläser, 1974 (8.6%; acc. no. AF328868). The pair-wise distance with *G. rugiensoides*, the species whose attachment hooks are morphologically similar to those of *G. salinae *n. sp., amounted to 14%. Of all available ITS rDNA sequences of *Gyrodactylus *(146 on GenBank), *G. salinae *n. sp. has the longest ITS1 fragment (774 bp), followed by *Gyrodactylus teuchis *Lautraite, Blanc, Thiery, Daniel *et *Vigneulle, 1999 (720 bp; acc. no. AJ249350). The ITS1 has a GC content of 42.1% and an imperfect repeat of an 8 bp motif (GAGAGAGT), starting at position 101 (copynumber 4.4). The ITS1 of *G. anguillae *and *G. micropsi *did not have any repeat element. The 5.8S rRNA gene is 158 bp long, which is 1 bp longer than all other *Gyrodactylus *species sequenced so far; the ITS2 (402 bp) has a median size.

**Table 2:** Pair-wise genetic distances based on the 5.8S and ITS2 rDNA fragment of *Gyrodactylus salinae *n. sp. and the *Gyrodactylus *species showing highest similarity in the BLAST search on GenBank (Tamura-Nei + gamma model)

*Gyrodactylus *species	1	2	3	4	5	6	7
1. *G. salinae *n. sp.							
2. *G. anguillae*	0.083						
3. *G. rugiensis*	0.135	0.139					
4. *G. micropsi*	0.086	0.088	0.113				
5. *G*. cf. *micropsi*	0.097	0.076	0.121	0.048			
6. *G. rugiensoides*	0.140	0.151	0.023	0.124	0.132		
7. *G. eyipayipi*	0.179	0.189	0.225	0.193	0.188	0.232	
8. *G. longidactylus*	0.112	0.098	0.128	0.075	0.091	0.137	0.191

### Comments

Morphologically, the opisthaptoral hooks, notably the marginal hook sickles, of *G. salinae *n. sp. are similar to those of *G. rugiensoides *described from the sand goby *Pomatoschistus minutus *(Pallas) collected from Texel, The Netherlands [22] (Figure 2d-g). Given the potential overlapping distribution of these two fish hosts within the Mediterranean Sea [1], it is important to detail the subtle features in hook morphology that permit their discrimination from one another. Two paratypes of *G. rugiensoides *were examined for morphological comparison with *G. salinae *n. sp. The paratypes were re-measured and the marginal hook sickle re-drawn and overlaid with *G. salinae *n. sp. for a direct comparison (Table 1; Figure 2e-g). There was a good agreement between the measurements obtained in the current study and those presented in Huyse & Volckaert [22]. The present study, however, provides an additional set of measurements taken from the type material of *G. rugiensoides *for direct comparison with the opisthaptoral features of *G. salinae *n. sp. Although the hamuli roots in both species narrow after their union with the shaft, giving the anterior edge of the dorsal bar attachment point a small but distinct edge, this appears to be more prominent in *G. rugiensoides *than on the hamuli of *G. salinae *n. sp. The ventral bar attachment points also differ; those of *G. rugiensoides *appear flat and rectangular, while those of *G. salinae *n. sp. are indented. The prominent ventral bar processes and the longer, slender ventral bar membrane of *G. salinae *n. sp. contributes to the discrimination of the two species. The similar morphology of the marginal hook sickles of both species though, requires careful examination. The union of the marginal hook shaft with the sickle divides the width of the sickle base into 3:2 (heel:toe) in both species, however, the sickle base is deeper in *G. rugiensoides *than in *G. salinae *n. sp. with a more angular, rectangular heel and a steeper faced, more robust toe (Figure 2f-g). The sickle shaft and sickle tip of *G. rugiensoides *is broader, proportionately so, than that of *G. salinae *n. sp. giving the latter the appearance of having a more open deeper sickle aperture. A comparison of soft body features suggests that the posterior pharynx bulb of *G. rugiensoides *(78.4 μm in diameter) is considerably larger than that of *G. salinae *n. sp. which measures in 24.6 μm in diameter. Although Huyse & Volckaert [22] described the MCO of *G. rugiensoides *as armed with one principal spine and five small spines, a closer examination of the paratypes shows that *G. rugiensoides *possesses nine small spines, as that of *G. salinae *n. sp. The MCOs of the two species are similar in length (*G. salinae *n. sp. 14.4 μm *vs G. rugiensoides *14.8 μm) but that of *G. rugiensoides *is slightly wider (12.7 *vs *15.1).

A molecular comparison of *G. rugiensoides *and *G. salinae *n. sp. showed that they were quite distinct. The pair-wise distance amounted to 14% (based on the 5.8S + ITS2 fragment). The ITS1 sequences were more difficult to align, due to length differences (up to 132 bp). Both species belong to the so-called marine *rugiensis*-group that also includes *G. anguillae*.

Additional sampling *A. fasciatus *from the Cervia Saline at several time points throughout the year, found that *G. salinae *n. sp. was present on their hosts in waters ranging from 35 ppt and 5°C during December to 65 ppt and 30°C during July in the wild, while under captive conditions, fish even maintained a low level of infection in freshwater (0 ppt) (pers. obs.).

## Discussion

*Gyrodactylus salinae *n. sp. is the first species to be formally described from *Aphanius fasciatus *and, also the first from the genus *Aphanius *Nardo, 1827. Over 420 species of *Gyrodactylus *have been described [19,20] and only six are known to parasitise cyprinodontids, all of them are recorded from *Cyprinodon *spp. (see Table 3). These are *G. cyprinodontis*, *G. nevadensis *and *G. saratogensis*, all from *Cyprinodon nevadensis nevadensis *Eigenmann *et *Eigenmann; *G. hargisi *and *G. mobilensis *from *Cyprinodon variegatus variegatus *Lacepède, and *G. tularosae *from *Cyprinodon tularosa *Miller *et *Echelle. No supporting molecular data, however, is available for any of these species. Morphologically, the marginal hook sickles of the *Gyrodactylus *species described from cyprinodontid hosts are markedly different (Figure 3). The marginal hook sickles of these species were re-drawn from the paratypes and holotypes, where available, and a comparison of their morphology with *G. salinae *n. sp. is given in Figure 3. The marginal hook sickles of *G. cyprinodontis*, *G. nevadensis *and *G. saratogensis *are morphologically very similar to one another (see Figure 3a, g, i). These three species were found on the same host, *Cyprinodon n. nevadensis*, and they differ in the size of their opisthaptor and its skeletal elements, notably the hamulus total length (*G. cyprinodontis *= 49 μm; *G. nevadensis *= 33 μm; *G. saratogensis *= 26 μm) which allows their ready discrimination from each other [23]. The opisthaptoral hooks of the type material of *G. cyprinodontis *and *G. nevadensis *were not completely flat and therefore reconstructed drawings of the marginal hook sickles of these two species were necessary and are presented here and compared with *G. salinae *n. sp. The marginal hook sickles of *G. hargisi*, *G. mobilensis*, *G. tularosae *and *G. salinae *n. sp. differ markedly in morphology from the other three species parasitising cyprinodontid hosts. When the gyrodactylid fauna on fish species belonging to the family Cyprinodontidae are considered and compared, then there are a number of species which possess similar marginal hook sickle morphologies. This has also been noted among species parasitising fish belonging to the pipefish family Syngnathidae, where the species *Gyrodactylus eyipayipi *Vaughan, Christison, Hansen *et *Shinn, 2010, *Gyrodactylus neretum *Paladini, Cable, Fioravanti, Faria *et *Shinn, 2010, *Gyrodactylus pisculentus *Williams, Kritsky, Dunnigan, Lash *et *Klein, 2008 and *Gyrodactylus shorti *Holliman, 1963, all possess a similar marginal hook sickle morphology [24]. The same is seen within species parasitising Poecilidae, whose gyrodactylids can be roughly allocated, based on their marginal hook sickle morphology, to one of three groups. The first group encompasses species with a large, approximately triangular sickle base and reduced sickle tips *i.e*. *Gyrodactylus bullatarudis *Turnbull, 1956, *Gyrodactylus costaricensis *Kritsky *et *Fritts, 1970, *Gyrodactylus jarocho *Rubio-Godoy, Paladini, García-Vásquez *et *Shinn, 2010 and *Gyrodactylus poeciliae *Harris *et *Cable, 2000. The second group of species which has large open faced sickles with a double angled sickle shaft and narrow sickle base *i.e*. *Gyrodactylus gambusiae *Rogers *et *Wellborn, 1965, *Gyrodactylus milleri *Harris *et *Cable, 2000 and *Gyrodactylus turnbulli *Harris, 1986. While the third group includes *Gyrodactylus rasini *Lucký, 1973 and *Gyrodactylus xalapensis *Rubio-Godoy, Paladini, García-Vásquez *et *Shinn, 2010 whose marginal hook sickles have rounded heels and approximately equal sized sickle shaft and point regions [25].

**Table 3:** *Gyrodactylus *species parasitising different family members belonging to the order Cyprinodontiformes

*Gyrodactylus *species	Recorded host species	Host family	References
G. avalonia *	*Fundulus diaphanus diaphanus *(Lesueur)	Fundulidae	[40]
*G. bulbacanthus*	*Fundulus zebrinus *(Jordan *et *Gilbert)	Fundulidae	[41]
*G. bullatarudis*	*Poecilia mexicana *Steindachner, *Poecilia reticulata *Peters, *Poecilia sphenops *Valenciennes, *Xiphophorus hellerii *Heckel, *X. hellerii *× *Xiphophorus maculatus *(Günther) hybrids	Poeciliidae	[25,42-44]
*G. costaricensis*	*P. sphenops*	Poeciliidae	[45]
*G. cyprinodonti*	*Epiplatys fasciolatus *(Günther)	Nothobranchiidae	[46]
*G. cyprinodontis*	*Cyprinodon nevadensis nevadensis *Eigenmann *et *Eigenmann	Cyprinodontidae	[23]
*G. cytophagus*	*Aplocheilichthys eduardensis *(David *et *Poll), *Aplocheilichthys normani *Ahl, *Aplocheilichthys pumilus *(Boulenger)	Poeciliidae	[46]
*G. funduli*	*Fundulus similis *(Baird *et *Girard)	Fundulidae	[47]
*G. gambusiae*	*Gambusia affinis *(Baird *et *Girard)	Poeciliidae	[48]
*G. hargisi*	*Cyprinodon variegatus variegatus *Lacepède	Cyprinodontidae	[49]
*G. jarocho*	*X. hellerii*	Poeciliidae	[25]
*G. lamothei*	*Girardinichthys multiradiatus *(Meek)	Goodeidae	[50]
*G. mexicanus*	*G. multiradiatus*	Goodeidae	[50]
*G. milleri*	*Poecilia caucana *(Steindachner)	Poeciliidae	[51]
*G. mobilensis*	*Cyprinodon v. variegatus*	Cyprinodontidae	[49]
*G. nevadensis*	*Cyprinodon n. nevadensis*	Cyprinodontidae	[23]
*G. pictae*	*Micropoecilia picta *Regan	Poeciliidae	[52]
*G. poeciliae*	*P. caucana*	Poeciliidae	[51]
*G. rasini*	*X. hellerii*	Poeciliidae	[53]
*G. recurvensis*	*Aplocheilus blockii *(Arnold), *Aplocheilus panchax *(Hamilton)	Aplocheilidae	[54]
*G. salinae *n. sp.	*Aphanius fasciatus *(Valenciennes)	Cyprinodontidae	current study
*G. saratogensis*	*Cyprinodon n. nevadensis*	Cyprinodontidae	[23]
*G. stegurus*	*Fundulus d. diaphanus*	Fundulidae	[55]
*G. stephanus*	*Fundulus grandis *Baird *et *Girard, *Fundulus heteroclitus heteroclitus *(L.)	Fundulidae	[55,56]
*G. tularosae*	*Cyprinodon tularosa *Miller *et *Echelle	Cyprinodontidae	[57]
*G. turnbulli*	*P. reticulata*, *Poeciliopsis *sp.	Poeciliidae	[43,58]
*G. xalapensis*	*Heterandria bimaculata *(Heckel)	Poeciliidae	[25]

* *Gyrodactylus avalonia *Hanek *et *Threlfall, 1969 is suspected to be a junior synonym of *Gyrodactylus arcuatus *Bychowsky, 1933, but until molecular characterisation of *G. avalonia *is available, this species is considered as valid (J. Lumme and S.D. King, *pers. comm*.).

A list of all the species of *Gyrodactylus *recorded from cyprinodontids and other fish hosts belonging to the order Cyprinodontiformes is presented in Table 3. In addition to these, there are a number of other "*Gyrodactylus*" species parasitising cyprinodontiform hosts that appear in the literature. These include *Gyrodactylus foxi *Rawson, 1973, *Gyrodactylus megacanthus *Wellborn *et *Rogers, 1967, *Gyrodactylus prolongis *Hargis, 1955, *Gyrodactylus stableri *Hathaway *et *Herlevich, 1973 and *Gyrodactylus trematoclithrus *Rogers, 1967, all of which were subsequently transferred to the genus *Fundulotrema *Kritsky *et *Thatcher, 1977 based on the presence of an additional peduncular bar [26].

The fact that the current species is (both morphologically and molecularly) more closely related to *Gyrodactylus *species parasitising marine gobies and eels than to those infecting cyprinodontids, might suggest that the ecology of the host, rather than host phylogeny plays an important role in this host-parasite system. Malmberg [10] found *G. anguillae *only on migrating elvers, which are relatively small and abundantly found in estuaries, as is the common goby *Pomatoschistus microps *(Krøyer), host to *G. micropsi*. There are other examples of ecological radiations onto distant-related hosts in *Gyrodactylus, e.g*. the *G. wageneri*-group primarily infects cyprinids but they are also found on sticklebacks, percids and cottids [27].

There is nothing known yet on the effect of *G. salinae *n. sp. on its host, but its closest relative so far, *G. anguillae*, has been reported as a pest in the culture of anguillid eels [28,29].

Based on the nuclear ITS fragment, the genus *Gyrodactylus *can be divided in two groups, one with a 'short' (347-473 bp) and one with a 'long' (535-688 bp) ITS1 fragment [30,31]. These ranges have recently been extended both to the lower end, with *Gyrodactylus cichlidarum *Paperna, 1968 having the shortest ITS1 sequence determined thus far (343 bp; acc. no. DQ124228), and *G. salinae *n. sp. having the longest ITS1 fragment (774 bp). The sequence of *G. salinae *n. sp. is also unique as the 5.8S gene is 158 bp long, whereas all other *Gyrodactylus *species so far had an invariant length of 157 bp. The new species clusters in the marine *rugiensis*-group which is part of the monophyletic 'long ITS' group, and appears most closely related to *G. anguillae*. Despite the morphological resemblance between *G. salinae *n. sp. and *G. rugiensoides*, they are genetically quite different (especially in the ITS1 fragment). This underlines again the fact that morphological and molecular evolution is not always correlated. The close affinity with *G. anguillae *and *Gyrodactylus *cf. *micropsi *Huyse, Audenaert *et *Volckaert, 2003 as based on the genetic distances does not necessarily equal sister species relationships. The true sister species of *G. salinae *n. sp. has probably not been sequenced yet since the difference between *Gyrodactylus *species from closely related goby species was for example much smaller (2-5%) [32] than the distance between *G. anguillae *and *G. salinae *n. sp.

## Conclusions

Although the specimens of *A. fasciatus *from Cervia Saline were moribund at the time of collection, this was not a consequence of the moderate *G. salinae *n. sp. infection but of the pool conditions (30°C, 65 ppt) from which they were collected which had a water depth of less than 15 cm and a heavy growth of algae. Given the ability of *G. salinae *n. sp. to tolerate a wide range of salinities and temperatures, it would appear that this species has the potential to readily adapt to the full spectrum of environmental conditions. What is unknown, however, is the potential of this species to survive on other neritic fish hosts when cohabiting isolated pools or interacting with other fish species in open, full strength seawater (*i.e*. 35 ppt). The potential utility of *Aphanius fasciatus *as a biological control against the larvae of the Asian tiger mosquito *Stegomyia albopicta*, in addition to the finding of a new parasite species, raises concerns regarding the health status of this host due to the parasitic infection. Further studies are required in order to define the potential role of *G. salinae *n. sp. as a pathogen, and the consequences that this finding might have on the use of indigenous populations of *A. fasciatus *as an alternative biological control to the use of various *Gambusia *species. The ecological risks of introducing *Gambusia *spp. to non-native environments has been stressed by many authors [33-35]. Cross breeding with indigenous species may lead to the extinction of the latter given that certain species have been shown to hybridise [36-38]. There are also the attendant risks of disease introduction linked with the movement of fish into new habitats [39]. The use of native fish, rather than introduced species, as biological controls, therefore, is highly recommended to avoid these potential ecological impacts.

In accordance with section 8.6 of the ICZN's International Code of Zoological Nomenclature, copies of this article are deposited at the following five publicly accessible libraries: Natural History Museum, London, UK; American Museum of Natural History, New York, USA; Museum National d'Histoire Naturelle, Paris, France; Russian Academy of Sciences, Moscow, Russia; Academia Sinica, Taipei, Taiwan.

## Competing interests

The authors declare that they have no competing interests.

## Authors' contributions

GP and APS conducted the morphological and morphometrical analyses, TH performed the molecular analysis. GP, APS and TH co-drafted the manuscript together. All authors read and approved the final version of the manuscript.
